# Hip-Joint Dislocation Reduced by Manipulation

**Published:** 1861-05

**Authors:** Z. P. Landrum

**Affiliations:** Columbus, Miss.


### *Hip-joint dislocation reduced by manipulation.* By Z. P. LANDRUM, M. D., Columbus, Miss.

DEAR DOCTOR:—I wish to direct the attention of the profession to Reid's plan of reducing hip-joint dislocations, by a brief report of a case which was entrusted to my care during the past year. I do this because I have recently met with a number of intelligent and respectable practitioners, who were not even aware of this great improvement in this department of surgery:

A negro man, belonging to Mr. Wm. T. Howard, of Oglethorpe Co., Ga., whilst engaged in hauling rails with an oxwagon, was buried beneath a load of this material by overturning his wagon on a hill-side. From this, beside extensive bruising of his flesh, there resulted a dislocation at the hip-joint, in which the head of the femur was thrown upwards and backwards, and rested on the dorsum of the ilium. When I reached my patient he had been removed to his cabin, was placed on his bed, and was suffering greatly. There was distortion of the hip, produced by the new position of the head of the femur, which could be readily felt, and recognised when moved; there was, of course, shortening of the limb, which though very perceptible, was not measured to ascertain its extent; the peculiar change in the position of the limb consisted in slight flexion of the thigh on the abdomen, the leg on the thigh, and considerable invertion of the foot and leg. A large number of our best practitioners, particularly in the country, would always prefer, under the old plan of treatment, to be relieved from the necessity of taking charge of this class of surgical cases. Because, for their relief, your patient must undergo a tedious, painful, and sometimes uncertain process of reduction, the details of which are not to all minds of easy recollection, nor to all hands of ready application.

In the last edition of Erichsen's Surgery, we find the following directions for the management of my case:

"The reduction of this form of dislocation is effected in the following manner: The patient having been put under the influence of chloroform, is laid on his back upon a strong table between two staples, one of which should be fixed to the floor, and another at a point above the level of the body, in a direct line with the axis of the limb, and about twelve feet apart. The counter extending force consisting of a jack-towel, or of a padded leather belt, must then be passed between the injured thigh and perineum, and fixed to the staple in the floor. The pulleys must now be attached to proper straps, or to a towel fixed with a claw-hitch knot, immediately above the knee, by one end; the other extremity being attached to the staple in the wall, which should be so situated as to be continuous with the axis of the lower part of the limb. The knee being then slightly bent and rotated inwards, traction is applied slowly and steadily until the head of the bone has approached the acetabulum, when the surgeon rotates the limb inwards so that the head may slip into its socket. If difficulty occur in raising the bone over the acetabulum, the plan recommended by Sir A. Cooper, of passing a towel under the thigh, to enable an assistant to lift the head of the bone over the brim of that cavity, may be had recourse to."

If the above plan of reduction is carried out, there is needed a jack-towel or padded leather belt, pulleys and straps, a strong table of proper height, two large staples, and several assistants. We cannot know that we have a dislocation of this character to deal with, until our patient has been examined, and it may be that when such an injury is found to exist, the circumstances of our patient may not furnish any single one of the needed expedients. If so, that prompt relief from the intense torture a case is sometimes suffering, must be delayed until a half of a neighborhood is hunted over in collecting pulleys, straps, staples, assistants, &c. Again, if the several requisite means are all at hand, and the physician understands exactly how to apply them, so as to make the increase in his extension with precise and well-timed gradations, and in fit and proper direction, the patient must suffer for hours, sometimes, the pain which so powerful an extending force must produce, when applied to the fibres of the several expanded layers of muscular tissue that resist the reduction; and they may not then yield, until he has undergone the debilitating process of blood-letting, combined with filthy nauseating doses of tartar emetic, and ipecac.

It is true, that Erichsen does condescend to notice our country-man's plan of reduction, in a little paragraph of five lines—a sort of lateral notice, a side wipe, such as an old woman's remedy for a severe malady might receive after full and explicit directions to try everything else first. So brief is the notice, that the ordinary reader of his book on this subject, who had not before heard of Reid's plan, would have his mind so pre-occupied with his plan of staples, straps and pulleys, that this strikingly brief, modest and unobtrusive notice of five lines would not make impression sufficient even to recur to it afterwards. Why this brevity? Why not contrast, at some length, an improvement of so much importance with the troublesome old plan he has taken pains to detail, and sanction with his deservedly high judgment? Why not tell the student that in this form of this dislocation he had always better try Reid's plan first, because he may and will, in nine cases out of ten, by it, reduce his dislocation without assistance, in a half minute; and, with a trifling amount of pain, lift his patient from a condition of exquisite torture to one of comparative ease and comfort? Why treat a discovery of so much importance, both to the patient and physician in these cases, with such indifference as barely to give it a passing notice?

Whatever answer there may be to these questions, I am satisfied that every one whose experience appreciates the merits of Reid's discovery, and knows the advantages it has over all other plans of reduction, and has read Erichsen's work on surgery, has had similar reflections. What then is

Reid's plan? Why, it is so simple that you will probably conclude that there is nothing in it—but in that you will be greatly mistaken.

As Abram (for I believe that was the boy's name) lay on the bed, I caught hold of his ankle, bent his leg on his thigh, carried the limb in this position through a circular sweep across the sound one, until I had bent it around as high as the pelvis, then, by abducting it slowly but directly outwards, the head resumed its position in the acetabulum immediately, and without the least difficulty. I felt it enter the socket with a slight jerk, and with such immediate relief to the negro that he exclaimed "it's all right now." To reduce this dislocation and relieve the negro of his intense pain did not require a half minute. I ask, if nothing is gained here in time, in suffering, in anxiety, in toil, to merit an earnest effort in impressing the importance of this discovery on the minds of the profession? I think it probable that in some forms of hip-joint dislocation there would be more difficulty than I experienced in this case in effecting the reduction by manipulations. But in the dislocation *upwards and backwards* there can be no great difficulty, with proper manipulations in succeeding in all cases. I mean when the head of the femur rests upon the dorsum Ilii. Recollect, that grasping the ankle and knee, the leg is to be fixed on the thigh, then carry the limb in this position across the sound one as high as the pelvis, then abduct the limb slowly, directly outwards, making, if necessary, slight movements of rotation. If this brief report shall cause some timid, cautious, doubting practitioner to adopt this plan in a case like unto mine, and meet with the same success, as I know he will, the writer will be amply compensated for this communication.

